# The Relationship between Complementary and Alternative Medicine Use and Breast Cancer Early Detection: A Critical Review

**DOI:** 10.1155/2012/506978

**Published:** 2012-12-19

**Authors:** Laura C. Dale, Carolyn C. Gotay

**Affiliations:** School of Population and Public Health, Faculty of Medicine, University of British Columbia, 2206 East Mall, Vancouver, BC, Canada V6T 1Z3

## Abstract

*Objective*. Complementary and alternative medicine (CAM) use is prevalent. Concurrently, breast cancer is the most common cancer in women worldwide, with early detection techniques widely available. This paper examined the overlap between participation in allopathic breast cancer early detection activities and CAM use. *Methods*. A systematic review examined the association between breast screening behaviors and CAM use. Searches were conducted on the PubMed, Embase, CINAHL, and NCCAM databases and gray literature between 1990 and 2011. STROBE criteria were used to assess study quality. *Results*. Nine studies met the search criteria. Four focused on CAM use in women at high breast cancer risk and five on average risk women. CAM use in women ranged from 22% to 82% and was high regardless of breast cancer risk. Correlations between CAM use and breast cancer early detection were not strong or consistent but significant relationships that did emerge were positive. *Conclusions*. Populations surveyed, and measures used to assess CAM, breast cancer screening, and correlates, varied widely. Many women who obtained allopathic screening also sought out CAM. This provides a foundation for future interventions and research to build on women's motivation to enhance health and develop ways to increase the connections between CAM and allopathic care.

## 1. Introduction

Complementary and alternative medicine (CAM) is defined as medical practices infrequently taught in medical schools nor widely available in hospitals, the latter being defined as “allopathic medicine” [1]. According to the National Center for Complementary and Alternative Medicine (NCCAM), CAM can be described using broad categories: that is, natural products, mind body practices, manipulative and body-based practices, and other approaches [2]. Over time, CAM practices may become accepted and integrated into allopathic medicine [2]. 

In a recent national survey of Americans, most people using CAM did so in complement with allopathic medicine [3]. CAM usage was positively associated with the number of personal health conditions and the number of doctor visits in the past 12 months. Only a small percentage used CAM to replace allopathic medicine and such “alternative medicine users” may have poorer health than complementary users [4]. In addition to many cultural factors contributing to variations in CAM use, it is important to better understand how CAM use and health practices influence and inform each other. 

A nationally representative study found that women in better health reported higher CAM use [5]. CAM users tend to have better health behaviors, with more physical activity, limited alcohol consumption, not smoking [6], and following a healthy diet [7], all of which are independently associated with CAM use. A survey of Medicare supplement plan enrollees found that 42% used CAM specifically for health improvements [8]. Since CAM users are highly involved in health practices, we hypothesized that they may also be more inclined to adhere to preventive strategies based in allopathic medicine, such as cancer screening. 

Breast cancer is the most commonly diagnosed cancer in women worldwide, with 1.38 million new cases and 458,000 deaths in 2008 [9]. Using early detection interventions, breast cancer can be diagnosed at an early stage when successful treatment is more likely. Multiple agencies and organizations around the world support mammography as the most reliable way to find breast cancer early, particularly in women 50 and older [10, 11]. A professional clinical breast exam (CBE) and “knowing one's breasts” or breast self-examination (BSE) are also recommended by some organizations [12, 13].

We were interested in learning whether participation in allopathic breast cancer early detection activities is associated with CAM use for women at both high and average risk of breast cancer. This paper provides a critical review of the literature to identify CAMs used, correlates of use, methodological strengths and weaknesses of the literature, and suggestions for future research and practice.

## 2. Methods

References were identified through PubMed and Embase database searches for 1990–2011. For PubMed, Mesh terms included “complementary therapies/utilization” AND “health behaviors,” and “breast neoplasms/prevention and control” AND “complementary therapies/utilization.” In Embase, similar search criteria were used with keywords including the explosion of “breast neoplasms” to include the “prevention” subheading AND the explosion of “complementary therapies” to include all subheadings. We also searched major Canadian government documents and other gray literature sources including Cumulated Index to Nursing and Allied Health Literature (CINAHL), the NCCAM website research database, and Google, and identified no additional papers. 

The Strengthening the Reporting of Observational Studies in Epidemiology (STROBE) statement was used as a guideline to ensure a high quality of research in this review [22]. A checklist of 22 items included indicators of study design, participant eligibility, variable assessment, potential bias, statistical methods, outcome data, and generalizability. Each paper was assessed according to these indicators and is fully available upon request. The STROBE statement was not used as tool to assess the methodological quality of the studies. 

We included all forms of CAM as described by the authors and excluded papers examining women currently or previously diagnosed with breast cancer as breast cancer screening recommendations differ vastly for these women. Use of self-reported or medical record-based mammography, BSE, and CBE were the indications of allopathic breast cancer early detection used here. 

 Results for CAM use and its association with breast screening behaviors are summarized separately for women at high and normal genetic risk for breast cancer. We support this separation as it has been suggested that many women at high-risk for breast cancer display signs of extreme cancer anxiety, leading to increased breast screening tendencies [23]. 

All studies included in the review were summarized using their description of the defined study population, participant response rate, data collection methods and analytic procedures.

## 3. Results

Table 1 summarizes nine studies (comprising 10 papers) that met the search criteria. Four studies focused on CAM use in women at high breast cancer risk (based on participation in a genetic testing or familial risk clinic or study), and five on average risk women. Most studies were based in the US, and two in Australia. Sample sizes ranged from a clinic-based sample of 104 [14, 15] to a large insurance claim database study with over 71,000 women [4]. A majority collected data through mailed questionnaires with response rates ranging 59%–86%. Eight of nine studies measured CAM through dichotomous responses regarding use of a series of CAM modalities. The exception was a records-based study where CAM use was extracted from claims data, which reported four CAM therapies paid for by the health insurance plan [18]. Numbers and types of CAMs queried varied considerably from one study to another, ranging from eight to 35. CAM content varied as well, with healthy diet considered a CAM in one study but not others. The period in which CAM use was queried ranged from “ever use” to “past year” with only one study assessing CAM use over two years [21]. Regarding breast screening, there was considerable variability in what was asked and the time frame used; mammography, CBE, and BSE were all frequently assessed. 

### 3.1. Prevalence and Types of CAM Use

Table 1 indicates a wide range of prevalence of CAM use, ranging from 8.3% [16] to 82% [18]. The lowest figure listed is difficult to interpret since it is based on the overall study sample, which included both men and women aged 18 and older [16]; no information was provided regarding CAM use in subpopulations comparable to most papers reported here: that is, middle aged women. CAM use in the four studies of women at increased breast cancer risk was 42% [14], 50% [20], 55% [17], 58% [15], and 69% (without prayer included) [19]. In studies that reported data specific to women, rates went from 22% [4] to 46% [7], 50% [20], and 82% [18]. The data indicate that CAM is used in a majority or a large minority of women regardless of risk status. Many women, in fact most, used more than one kind of CAM. Field et al. [17] reported that one woman used 26 different kinds of CAM.

Table 2 provides a summary of the kinds of CAMs reported. The range is broad and includes the full range of approaches delineated by NCCAM. Each study, however, assessed only a subset of the total therapies possible. The most commonly queried therapies were acupuncture, massage therapy, and meditation. CAM definitions used varied, with most studies omitting healthy eating and nutrition or specific diets. The controversial role of prayer as a CAM was highlighted in one study [19], which computed CAM rates with and without prayer, given its high endorsement as part of everyday life. 

CAM use was not assessed in a consistent way. The time frame for CAM use varied, from over the past month, to within the past year, to the past two years. Often CAM use was assessed through recall or prompting, with respondents asked to select those that they have used in the given time frame. In contrast, DiGianni's studies [14, 15] required participants to recall the types of CAM used from memory, with memory recalls aided by suggestions of major CAM categories. It is unclear how the assessment approach may have affected responses.

### 3.2. Is CAM Use Associated with Screening Behaviors for Breast Cancer?

#### 3.2.1. Women at High Risk of Breast Cancer

Of the four studies in women at increased breast cancer risk, two found no statistically significant association between CAM use and BSE [14] or mammograms [17]. Myers et al. [20] found statistically significant positive relationships between CBE, BSE, and mammography in univariate analyses, which disappeared after taking account of covariates in multivariate analysis. A fourth paper found a weak but statistically significant inverse relationship between BSE frequency and CAM use, such that women performing self exams less than once a month were more likely to use CAM [19]. 

#### 3.2.2. Women at Average Risk of Breast Cancer

Five studies examined women at non-increased breast cancer risk, and four of these assessed mammography. Two studies reported positive associations between CAM and mammography [4, 7], whereas two studies [16, 18] found no relationship. Downey et al. found that naturopathy had a significant negative association with mammography, while massage had a significant positive association. This study also looked at alternative therapy use—that is, using CAM rather than allopathic medicine during the period of observation. The researchers found that women who used CAM as well as biomedical care (i.e., complementary therapy users) were more likely to have a mammogram, whereas those who used CAM as an alternative and did not see a physician, were less likely to obtain mammographic screening [4]. 

One study found that herbal therapies and nutritional approaches but not phytoestrogens, were significantly correlated with BSE over the previous two years [18]. 

Two studies focused on CBEs. Gollschewski et al. [18] found no relationship between CAM and CBE, whereas Druss and Rosenheck [16] found that CAM users were more likely to receive CBEs.

### 3.3. Correlates of CAM Use

Assessing correlates of CAM use was limited since some studies reported only relationships for the overall study population, whereas others focused on a specific target group. Nonetheless, certain trends stand out. Eight of nine studies found that higher education was linked with more CAM use [4, 7, 16–21] and most found that higher CAM use was linked with being younger [4, 7, 18, 21] and having better health behaviors [7, 15, 17, 18, 20]. These findings are consistent with other CAM literature [24]. Higher CAM use was linked with higher anxiety or worry in several studies [15, 17, 19] and with a lower perceived breast cancer risk [15, 17]. 

## 4. Discussion

This paper reviewed the literature on CAM use in women participating in early detection for breast cancer. The studies were high quality in terms of defining their study populations, response rates (all reported response rates of 59% or greater), well-defined data collection methods, and analytic procedures that used both univariate and multivariate strategies.

We identified nine studies that reflected a range of populations and assessment techniques. While the heterogeneity of the research makes drawing firm conclusions difficult, some findings are of particular interest. CAM use is common among women, regardless of risk status. Congruent with previous research, those who relied solely on CAM therapies as an alternative to conventional medicine were less likely to obtain mammograms whereas women who used CAM as a complement to allopathic medicine were more likely to be screened. Of particular interest is the positive association found for women who used massage therapy. Many barriers have been identified, including feelings of embarrassment or modesty, which prevent women from receiving breast exams [25]. Massage therapy could be a positive way to decrease these barriers, as individuals receiving massage had a higher body image perception, possibly due to positive effects of being physically touched [26]. This demonstrates the possible ways that CAM and allopathic medicine could complement one another and increase the odds that a woman will feel comfortable receiving a mammogram and will seek one out.

Literature in the area of soy consumption and breast cancer is controversial and to our surprise, seven studies did not directly assess soy intake. In higher soy-consuming cultures, mammographic densities have been positively related to soy intake [27] and dense breast tissue poses difficulties for effective mammographic breast cancer screening. It would have thus been important to have had additional data on the soy-breast screening relationship as high soy consuming women may have added benefits from increased breast screening. It is recognized that soy consumption, alongside other CAM practices, could have been captured in some questionnaires through “long answer” or “other” questions. It is positive that the findings from this review suggest that women with higher CAM use also have higher rates of breast screening procedures. Much more research in the area of breast screening and CAM soy use is needed to verify these associations. 

Several methodological concerns need to be considered. These studies were almost all cross-sectional, making it impossible to determine a causal relationship between CAM use and breast screening. Although not all studies found CAM use and breast early detection use were correlated, significant relationships that did emerge were positive. However, this leaves the question of whether higher rates of breast screening result from the holistic and preventive focus of CAM; or whether the individuals who are interested in prevention and early cancer detection are more likely to use CAM; or whether both use of CAM and breast early detection modalities are due to another causal factor, such as self-efficacy for health. Developing and testing conceptual models in this area is a key research priority.

Assessing CAM accurately and consistently is challenging. As seen in Table 2, there was considerable variability among the studies in terms of types of CAM use assessed. In all but one study, CAM use was based on self-reports. The largest study used a sample of over 71,000 women had the advantage of drawing on objective claims reports of services billed to the health insurer which are not subject to recall or social desirability bias; however, only a limited number of CAMs were listed which precluded comparisons with other reports [4]. 

Another challenge when comparing CAM research papers is failure to ascertain CAM duration, frequency and dose. For example, a woman who partakes in a yoga session every month for an hour is likely to exhibit different health qualities than another who partakes in a 90-minute session each morning. Frequency and intensity of CAM use may be important to assess. 

Researchers who assess nutrition and physical activity are familiar with the difficulties associated with measurement of lifestyle variables. Major advances have been made in these research areas through the introduction of standardized assessment tools that include food frequency and physical activity questionnaires. CAM research would benefit significantly from the introduction of standardized and validated questionnaires that would allow comparisons over time and across studies.

## 5. Conclusions

To our knowledge, this is the first review paper examining CAM use and its association with breast cancer screening. Although a majority of women use CAM therapies, and most women also participate in breast cancer early detection, there has been little attention to the overlap between these two phenomena, and the potential for one set of health behaviors to inform the other.

We found a wide variety of findings in populations assessed, and measures used to assess CAM, breast cancer screening, and correlates thereof. Some findings stand out such as the high use of CAM in general and the fact that when there is a significant relationship between CAM use and breast cancer early detection, it tends to be a positive relationship; in other words, women who are motivated to obtain allopathic screening are also motivated to seek out other ways to care for themselves. They tend to be more educated and in better health, and to exhibit better health behaviors, and as such, are availing themselves of a wide range of preventive care. This provides a foundation for future interventions to increase the connection between CAM and allopathic providers, to build on the strengths of what each can offer, and to maximize on patient motivation and preferences to increase breast health and reduce breast cancer risk. Research is needed to make this potential a reality.

## Figures and Tables

**Table 1:** Summary of Studies.

Study^a^ (Year)	Study design	Sample size	Selected participant characteristics	Data collection methods and CAM assessment	Period of CAM use assessed	Proportion of cancer-free participants using CAM	Breast screening measures^b^	Relevant findings	Correlates of more CAM use
DiGianni, (2003 and 2006), [14, 15]	Cross-sectional (2003) Prospective cohort (2006)	104 without cancer history	>18 yrs, F; Enrolled in a breast/ovarian genetic testing clinic; USA	Mailed questionnaire 83% response rate at 1 year Y/N 8 CAMs^c^	Ever use	*Baseline* 42%; 33% used 1-2 CAMs *1 yr followup* 58%	*Baseline* BSE (rarely/often) *1 year followup* CBE (# in past year) Mam BSE	*Baseline*—no associations *1yr followup* CBE negatively correlated with # of CAMs used at 1 year (*P* < 0.004); No association for Mam or BSE	*Baseline* Perceived cancer risk, sunscreen use, fruit/vegetable consumption *1 yr followup* Higher anxiety, lower perceived breast cancer risk

Downey, (2009), [4]	Cross-sectional	71,083	52–64 yrs, F; enrolled in two washington state insurance companies; USA	Insurance claims data 4 kinds of insurance-paid CAM	Past year	Approximately 22–26% used CAM (depending on year); average 8 visits/yr <1% used only CAM therapies	Mam (past 2 years)	Complementary CAM use more likely to have Mam (OR 1.044; *P* = 0.031); alternative CAM less likely to have Mam (OR 0.006; *P* = 0.000); naturopathy negatively associated with Mam (OR 0.736; *P* = 0.000); massage positively associated with Mam (OR 1.196; *P* = 0.000)	Younger age, higher disease burden, enrolled in fee-for-service products; over the 3 measurement years; areas with lower education, income, and percentage of minority residents

Druss, (1999), [16]	Cross-sectional	10,675 overall (# answering breast screening items NR^f ^)	>18 yrs, M/F, age and sex-appropriate subset answered breast questions; national probability sample (medical expenditure panel survey); USA	Interview 77% response rate Y/N 13 CAMs that are practitioner-based	Past year	8.3% of overall sample; NR^f^ for women answering breast screening items	CBE Mam (past year)	More CBE in CAM users (58.7%–95% CI: 57%–60%) than non-users (69.7%–95% CI; 65%–74%) (*P* < 0.001)^d^ No association with Mam	Female, caucasian, higher education, and residing in the west (USA) (only reported for overall sample)

Field, (2009), [17]	Cross-sectional	892	62% 40 yrs+, F; enrolled in the high breast cancer risk cohort; Australia and New Zealand	Mailed questionnaire— 73% response rate Y/N 35 CAMs	Ever use	*General Use* 55%; 80% >1 CAM therapy, 30% >4 CAM therapies; *Intention to prevent cancer* 6% of participants	Mam (past 3 yrs)	No association	More education and physical activity, clinical anxiety, being a former smoker and lower perceived BC risk

Gollsche-wski, (2005), [18]	Cross-sectional	886	48–67 yrs, F; 61%, <55 yrs; random sample south-east Queensland, Australia	Mailed questionnaire— 59% response rate Y/N questions on herbal, phytoestrogen, nutrition and supplement CAMs	Ever use	82% 67% used nutritional approaches, 56% used phytoestrogens, 41% used herbal therapies	CBE, BSE (past 2 yrs)	More BSE in herbal therapy users (OR 1.69, 95% CI 1.34–2.52; *P* = 0.01); and nutritional users (OR 1.68, 95% CI 1.13–2.50; *P* = 0.01); no association with CBE	Younger, higher education, middle income, lower smoking, previous hormone therapy, good physical/general health

Gray, (2002), [7]	Cross-sectional	4404	>40 yrs, M/F; stratified sample (by chronic conditions) from health plan; Minnesota, USA	Mailed questionnaire— 86% response rate Y/N 17 CAMs	Past year	42% overall; 46% F	Mam (past yr)	CAM users significantly more likely to have had Mam (67% versus 62%)	Female, younger, higher education, single, employed, health limitations, improved health over past year. More exercise, vegetable intake, fast food consumption; less dietary fat and alcohol (only reported for overall sample)

Mueller, (2008), [19]	Cross-sectional	135 without cancer history, knew BRCA1/2 status	25–56 years of age, F; Enrolled in high genetic breast cancer risk clinic; USA	Telephone interview Y/N 13 CAMs	Past year	78% overall; 69% if spiritual healing/prayer are excluded^e^; average 2.3 CAM therapies; 34% ≥3 CAM therapies (overall sample)	Mam (annual) BSE (Monthly)	BSE and CAM use inversely related (OR 0.3, 95% CI 0.1–0.8; *P* = 0.017); no association with Mam	Older, higher education, ovarian cancer worry

Myers, (2008), [20]	Cross-sectional	2,198, varying risk based on family history	Average 63 yrs, F; family members of women enrolled in breast cancer family study; USA	Mailed questionnaire—70% response rate Y/N 8 CAMs	Ever use	*Intention: Preventing Cancer* 50%; 42% used 1 CAM, 32% used 2 CAMs, 15% used 3, and 12% used >3 CAM therapies.	BSE, CBE, Mam (ever)	In the univariate analysis, all 3 breast behaviors were associated with CAM use (OR 1.33, 95% CI 1.15–1.54; *P* = 0.0002); In the multivariate analysis, associations did not remain significant.	Higher education, general health behaviors, optimism (multivariate analyses)

Robinson, (2002), [21]	Cross-sectional	1,593	>18 yrs, M/F, attendees at health fair USA	Questionnaire Y/N 8 CAMs, 13 herbs	Past 2 years	68%; 63% used herbs/supplements	CBE Mam (Past 2 years)	No association	Younger, female, higher education (high school completion), lower levels of health insurance (only reported for overall sample)

^a^Studies listed by first author.

^b^CBE: clinical breast examination; Mam: mammography; BSE: breast self-examination.

^c^Y/N refers to dichotomous responses to use of each CAM treatment.

^d^These data reflect the authors' abstract, data section, and conclusions; the table in the paper presents opposite numbers and is assumed to be a typesetting error.

^e^Participants with cancer were included in this calculation because the authors state that overall patterns of the CAM therapies used didn't differ between cancer survivors and women without cancer and data were not presented separately for each group.

^f^NR: No response.

**Table 2:** Complementary and Alternative Therapies as Reported by Selected Studies.

	Study								
	(1)	(2)	(3)	(4)	(5)	(6)	(7)	(8)	(9)
Natural products and diet									
General vitamins/supplements	x	x		x					
Chondroitin							x		
coQ10				x					
Creatin							x		
Glucosamine							x		
Omega-3 folic acid		x							
High dose megavitamins							x		x
Selenium		x							
Vitamin E					x				
Other vitamins/supplements		x							
General herbal remedies	x		x						x
Bee Pollen							x		
Black Cohosh					x				
Dong Quai					x				
Echinacea							x		
Essiac				x					
Evening Primrose oil					x				
Flaxseed		x							
Ginko							x		
Ginseng					x		x		
Green tea		x							
Kava Kava							x		
Milk thistle							x		
Red clover					x				
Saw Palmetto		x					x		
Shark cartilage		x		x					
Saint John's Wort				x			x		
Soy		x		x					
Valerian							x		
Wild Yam					x				
Other herbs		x							
Hormones									
Herbal Rx for menopause		x							
Phytoestrogen supplements					x				
Dietary phytoestrogens					x				
Melatonin		x					x		
Tropical progesterone cream					x				
Special diet	x		x						
Commercial weight loss programs									x
Healthy eating					x				
Low fat diet		x							
Macrobiotic		x							
Soy rich diet		x							
Vegan		x							
Vegetarian		x							
Organic products				x					
Lifestyle diets									x
Nutritional supplements					x				
Other diets		x							

Manipulative and body-based practices									
Massage therapy	x	x	x	x		x	x	x	
Reflexology		x							

Mind and body medicine									
Acupuncture	x	x	x			x	x	x	x
Acupressure	x								
Hypnosis			x				x	x	x
Imagery/visualization		x	x					x	x
Meditation	x	x	x	x				x	
Relaxation techniques			x	x				x	x
Tai chi/Chi gong		x							
Yoga	x	x	x	x					
Other mind body		x							

Consultations									
Counselor/psychologist		x							
Chiropractic therapy				x		x	x	x	x
Dietician		x							
Herbalist								x	
Homeopathy		x	x				x	x	x
Lifestyle advice								x	
Naturopath		x				x	x		
Nutritional advice								x	
Osteopathy		x				x			

Energy therapies	x		x						x
Reiki		x							
Biofeedback/Energy healing		x	x				x	x	x

Whole medical systems									
Traditional Chinese medicine		x						x	
Ayurveda								x	
Folk remedies									x
American Indian								x	

Others			x	x				x	
Exercise	x								
Prayer/spiritual practices		x	x	x				x	x
Support groups				x					x
Other physical therapies		x							

Studies: (1) DiGianni et al., (2003 and 2006), [14, 15]; (2) Field et al., (2009), [17]; (3) Mueller et al., (2008), [19]; (4) Myers et al., (2008), [20]; (5) Gollschewski et al., (2005), [18]; (6) Downey et al., (2009), [4]; (7) Robinson et al., (2002), [21]; (8) Druss and Rosenheck (1999), [16]; (9) Gray et al., (2002), [7].
